# Can neuroprotection effectively manage primary open-angle glaucoma? a protocol of systematic review and meta-analysis

**DOI:** 10.1097/MD.0000000000020380

**Published:** 2020-06-05

**Authors:** Yan-xiu Qi, Jian Zhang, Xing-jie Su

**Affiliations:** Department of Ophthalmology, First Affiliated Hospital of Jiamusi University, Jiamusi, China.

**Keywords:** efficacy, neuroprotection, primary open-angle glaucoma

## Abstract

**Background:**

This study aims to assess the efficacy of neuroprotection (NP) for the management of patients with primary open-angle glaucoma (POAG).

**Methods:**

A comprehensive search will be carried out from the beginning to the February 29, 2020 in the electronic databases: Scopus, Web of Science, PUBMED, EMBASE, Cochrane Library, Cumulative Index to Nursing and Allied Health Literature, WANGFANG, and China National Knowledge Infrastructure. There are no limitations related to the language and publication date. Two researchers will independently undertake study selection from searched literatures, extract data from included trials, and appraise study quality using Cochrane risk of bias tool. Any disagreements will be solved by a third researcher through consultation. RevMan 5.3 software will be employed for statistical analysis.

**Results:**

This study will provide a high-quality synthesis of randomized controlled trials of NP for the management of patients with POAG.

**Conclusions:**

The results of this study will help to create proposals for the treatment of POAG using NP.

**Systematic review registration:**

INPLASY202040107.

## 1 Introduction

Primary open angle glaucoma (POAG) is a chronic and progressive optic neuropathy, which is 1 of the leading causes of blindness.^[1–3]^ It is characterized by the acquired optic nerve atrophy, loss of ganglion cells and axon, visual field deterioration with open angles when intraocular pressure is elevated, and mostly affects people over 40 years old.^[4–9]^ Despite thousands of studies has investigated the POAG, its pathogenesis is still unclear.^[10–13]^ Thus, effective treatments are very important as early as possible.

A variety of studies have shown the presence of neuroprotection (NP) for the management of POAG.^[14–21]^ However, their results are still contradictory. Thus, the objective of current study will summarize current data from primary trials to assess the efficacy of NP for the management of patients with POAG.

## 2 Methods

### 2.1 Study registration

This study has been funded and registered through INPLASY202040107. It has been performed follows the Preferred Reporting Items for Systematic Reviews and Meta-Analyses Protocols guideline.^[22,23]^

### 2.2 Inclusion criteria for study selection

#### 2.2.1 Types of studies

All potential randomized controlled trials (RCTs) that utilizing NP for the treatment of patients with POAG will be included in this study. Any other studies, except the RCTs will be excluded.

#### 2.2.2 Types of participants

Any patients who were clinically diagnosed as POAG will be included in spite of their nationality, race, sex, age, and duration of POAG.

#### 2.2.3 Types of interventions

All patients in the experimental group received NP intervention alone for their treatment.

All patients in the control group could receive any treatments, such as surgery.

#### 2.2.4 Types of outcome measurements

##### 2.2.4.1 Primary outcomes

Visual acuity,

Contrast sensitivity.

##### 2.2.4.2 Secondary outcomes

Visual fields,
Intraocular pressure,
Blood pressure,
Severity of oxidative stress,
Antioxidant defense,
Bioelectric activity of the retina,
Total sensitivity of the retina for central field of view,
Blood flow to the eye and optic nerve head,
Rate of progression of glaucoma, and
Incidence of adverse events.

### 2.3 Information sources and search procedure

#### 2.3.1 Search electronic databases

A comprehensive search procedure will be performed from the inception to the February 29, 2020 from the following electronic databases: Scopus, Web of Science, PUBMED, EMBASE, Cochrane Library, Cumulative Index to Nursing and Allied Health Literature, WANGFANG, and China National Knowledge Infrastructure. No language and publication date limitations will be applied to any electronic databases. An overview of search strategy for PUBMED is created (Table 1). Identical search strategies for other electronic databases will be modified and applied.

**Table 1:**
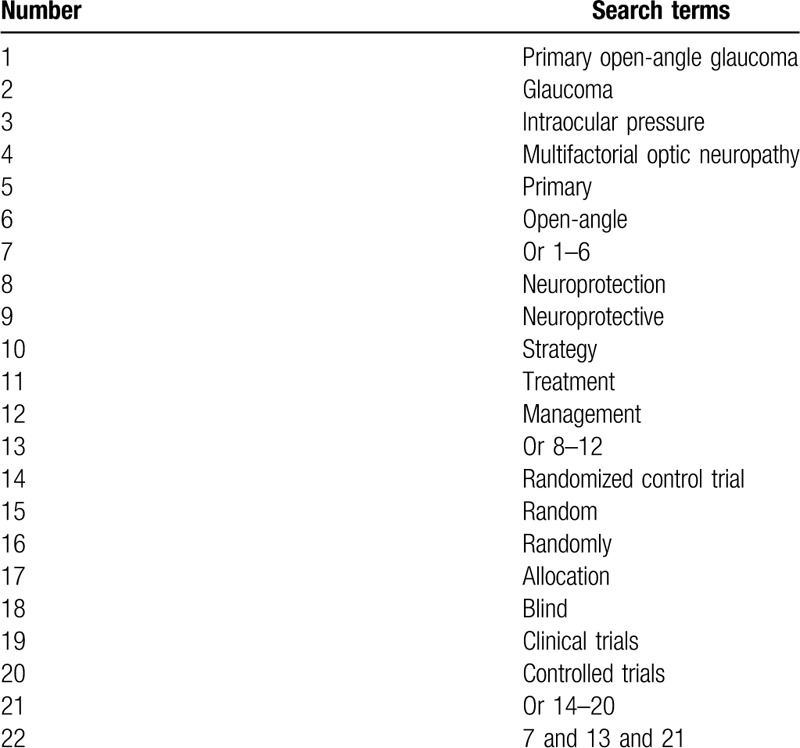
Search strategy sample for PUBMED.

#### 2.3.2 Search for other resources

In addition to the electronic databases, we will search clinical trial registries, conference proceedings, Google Scholar, and reference lists of relevant reviews.

### 2.4 Study selection and data extraction

#### 2.4.1 Study selection

Titles/abstracts of all searched literatures will be scanned by two independent authors after duplications have been removed using EndNote 7.0 software, and all unrelated studies will be deleted. Then, same two authors will carefully read full papers of the remaining studies against all inclusion criteria, and all eligible trials will be decided to be included. In the case of differences between two authors, a third author will be invited to discuss them and a consensus will be reached after discussion. The process of study selection will be presented in a flow chart.

#### 2.4.2 Data extraction and management

All information will be extracted from each eligible trial by 2 independent authors using predefined standard data extraction sheet after an initial pilot with three included studies. If any divergences occur between two authors, another experienced author will be invited to help solve them by discussion. The extracted data form includes title, first author name, publication time, journal, country, inclusion and exclusion criteria, diagnostic criteria, sample size, patient characteristics (such as age, gender, disease duration and severity), research setting and design, details of interventions and controls, outcome indicators, safety, results, conclusions, follow-up data, and other related information.

#### 2.4.3 Dealing with insufficient or missing data

Any insufficient or missing information will be obtained by contacting primary corresponding authors using email. If those data is not available, we will analyze the obtainable data only, and will discuss its potential impacts to the study findings.

#### 2.4.4 Trial quality assessment

The methodological quality of each trial will be independently assessed by 2 authors utilizing Cochrane Risk of Bias Tool for RCTs specifically. This tool has 7 parts, and three different grades (high, unclear, and low risk of bias) will be employed to check each item. Any discrepancies will be settled by another experienced author through discussion.

### 2.5 Statistical analysis

#### 2.5.1 Data synthesis

We will undertake statistical analysis using RevMan 5.3 software. Dichotomous data, such as rate of progression of glaucoma will be calculated as risk ratio and 95% confidence intervals. Continuous data, such as visual acuity and contrast sensitivity will be presented as mean difference or standardized mean difference and 95% confidence intervals. We will examine statistical heterogeneity using *I*^2^ statistic test. *I*^2^ ≤50% means low level of heterogeneity, and a fixed-effects model will be applied. When necessary, we will carry out meta-analysis if it is possible. *I*^2^ > 50% suggests high level of heterogeneity, and a random-effects model will be exploited. In addition, we will run subgroup analysis to explore possible sources of obvious heterogeneity. We can not find out such sources, a meta-analysis will not be performed. Instead, we will carry out a narrative synthesis of outcome data.

#### 2.5.2 Unit of analysis

If cross-over trials are eligible, we will only extract and analyze outcome data from the first study period.

#### 2.5.3 Subgroup analysis

A subgroup analysis will be performed to identify the causes of significant heterogeneity based on the variations in study characteristics and population characteristics, and differences in interventions and comparators, and outcomes.

#### 2.5.4 Sensitivity analysis

A sensitivity analysis will be investigated to check the stability and robustness of the study findings by removing trials with high risk of bias.

#### 2.5.5 Reporting bias

If more than 10 trials are included, we will examine the reporting bias by a funnel plot and Egger linear regression test.^[24,25]^

#### 2.5.6 Overall quality of evidence

Two independent authors will test the quality of evidence for each major outcome indicator using Grading of Recommendations Assessment Development and Evaluation.^[26]^ Any conflicts will be disentangled by another experienced author through consultation.

### 2.6 Dissemination and ethics

We will publish this study on a peer-reviewed journal or a conference meeting. This study will not need ethic approval, because it will not analyze individual patient data.

## 3 Discussion

Currently, there is a need for a summary of the evidence about the efficacy of NP for the treatment of patients with POAG. However, at literature level, there is no systematic review focusing on this point. This study will perform study selection and data extraction according to the identification of published and unpublished studies, and we will carry out assessment of study's methodological quality. The findings of this study will provide a clear summary of the evidence on NP for patients with POAG, which may supply highlights and reference for the strategies of clinical practice.

## Author contributions

**Conceptualization:** Yan-xiu Qi, Jian Zhang, Xing-jie Su.

**Data curation:** Yan-xiu Qi, Jian Zhang, Xing-jie Su.

**Formal analysis:** Yan-xiu Qi, Xing-jie Su.

**Funding acquisition:** Jian Zhang.

**Investigation:** Jian Zhang.

**Methodology:** Yan-xiu Qi, Xing-jie Su.

**Project administration:** Jian Zhang.

**Resources:** Yan-xiu Qi, Xing-jie Su.

**Software:** Yan-xiu Qi, Xing-jie Su.

**Supervision:** Jian Zhang.

**Validation:** Yan-xiu Qi, Jian Zhang, Xing-jie Su.

**Visualization:** Yan-xiu Qi, Jian Zhang, Xing-jie Su.

**Writing – original draft:** Yan-xiu Qi, Jian Zhang, Xing-jie Su.

**Writing – review and editing:** Yan-xiu Qi, Jian Zhang, Xing-jie Su.
